# Validation of Taylor’s Frozen Hypothesis for DAS-Based Flow

**DOI:** 10.3390/s25133840

**Published:** 2025-06-20

**Authors:** Shu Dai, Lei Liang, Ke Jiang, Hui Wang, Chengyi Zhong

**Affiliations:** National Engineering Research Center of Fiber Optic Sensing Technology and Networks, Wuhan University of Technology, Wuhan 430070, China; 290303@whut.edu.cn (S.D.); k.jiang@whut.edu.cn (K.J.); wanghui1989@whut.edu.cn (H.W.); 346626@whut.edu.cn (C.Z.)

**Keywords:** circular pipe turbulence, Taylor’s frozen hypothesis, distributed optical fiber acoustic sensing, dispersion feature enhancement, non-intrusive flow measurement

## Abstract

Accurate measurement of pipeline flow is of great significance for industrial and environmental monitoring. Traditional intrusive methods have the disadvantages of high cost and damage to pipeline structure, while non-intrusive techniques can circumvent such issues. Although Taylor’s frozen hypothesis has a theoretical advantage in non-intrusive velocity detection, current research focuses on planar flow fields, and its applicability in turbulent circular pipes remains controversial. Moreover, there is no precedent for combining it with distributed acoustic sensing (DAS) technology. This paper constructs a circular pipe turbulence model through large eddy simulation (LES), revealing the spatiotemporal distribution characteristics of turbulent kinetic energy and the energy propagation rules of FK spectra. It proposes a dispersion feature enhancement algorithm based on cross-correlation, which combines a rotatable elliptical template with normalized cross-correlation coefficients to suppress interference from non-target directions. An experimental circulating pipeline DAS measurement system was set up to complete signal denoising and compare two principles of flow velocity verification. The results show that the vortex structure of turbulent flow in circular pipes remains stable in the convection direction, conforming to theoretical premises; the relative error of average flow velocity by this method is ≤3%, with significant improvements in accuracy and stability in high-flow zones. This study provides innovative methods and experimental basis for non-intrusive flow detection using DAS.

## 1. Introduction

In the fields of industrial and environmental monitoring, the accurate measurement of pipeline flow is a crucial aspect for ensuring system operational efficiency, rational resource allocation, and safety control. Traditional measurement methods [1] typically require physical modifications to the pipeline or installation of specialized equipment, which not only increases construction costs but may also affect the structural integrity and operational stability of the pipelines. In contrast, non-invasive pipeline flow measurement technology can acquire flow data without altering the original structure of the pipeline, offering greater flexibility and applicability. This technology indirectly analyzes fluid motion characteristics through physical principles such as acoustic waves, electromagnetic induction, or thermodynamics, thereby deriving flow parameters [2]. This measurement method reduces maintenance frequency and downtime, lowers operational costs, and enhances the overall reliability of the system, serving as an important support means for achieving efficient, safe, and sustainable operations in modern industrial systems.

Distributed Acoustic Sensing (DAS) technology has become a research hotspot in the field of non-invasive pipeline flow measurement due to its unique spatial continuous monitoring capability. Its core principle is that when the fluid is in a turbulent state, vortex motion-induced pressure fluctuations are transmitted outward through the pipe wall, causing dynamic strain on the fiber optic attached to the outer wall of the pipeline. The DAS system detects phase changes in backscattered Rayleigh light to obtain distributed dynamic strain along the pipeline. By analyzing the mapping relationship between this strain signal and fluid velocity, non-invasive flow measurement of the pipeline can be achieved. There are three main technical approaches for flow inversion methods based on pipeline vibration signals. The first category is based on the principle model of Flow-Induced Vibration (FIV) [3], which establishes empirical correlations with flow velocity using statistical measures such as variance and power spectral density from single-point vibration signals. However, the randomness of turbulent pulsations leads to the requirement of long-term averaging for stable statistical features, severely limiting real-time measurement capabilities. Additionally, FIV models utilize local measurement point vibration information and fail to effectively integrate distributed measurement data obtained by the DAS system along the axial direction of the pipeline. The second category is the wave velocity analysis method based on the acoustic Doppler effect [4,5]. It performs a two-dimensional Fourier transform on the two-dimensional spatiotemporal vibration signals collected along the pipeline and identifies the acoustic wave velocities propagating in the downstream and upstream directions from the FK spectrum, calculating flow velocity through their difference. Although this model fully utilizes the distributed sensing capabilities of DAS, it requires throttling devices such as valves and orifice plates to generate strong acoustic emission sources in engineering applications. This active disturbance to the flow field essentially constitutes an invasive intervention into the measured system, going against the principle of non-invasive measurement and limiting its application under steady flow conditions. The third method is based on Taylor’s frozen turbulence hypothesis [6], assuming that turbulent vortices maintain structural stability along the convection velocity direction before dissipation. Cross-correlation is used to calculate the time delay between multiple adjacent measurement points to determine vortex migration speed, thereby inverting average flow velocity. This method does not require an external excitation source and can fully leverage the advantages of DAS’s distributed measurement capabilities, making it theoretically the ideal solution for non-invasive measurements.

However, although the Taylor frozen hypothesis model demonstrates significant technological advantages, there are currently no precedents for applying Taylor’s frozen hypothesis to DAS-based non-invasive pipeline flow measurement in existing studies. Moreover, current research on Taylor’s frozen hypothesis is primarily based on planar flow fields [7], and its conclusions’ applicability in complex circular pipe turbulence remains controversial. These controversies mainly stem from two aspects: one is the geometric characteristics of pipeline flow, such as the axial symmetry and circumferential flow patterns of the circular pipe, which make the flow structure more complex [8]; the other is the non-local nature of pressure fluctuations, meaning that pressure changes are affected not only by local flow but also closely related to the entire flow field’s velocity distribution through fluid dynamics equations [9].

To address the above issues, this paper will simulate the turbulent flow field of the pipeline through a large eddy simulation numerical model to verify the effectiveness of Taylor’s frozen hypothesis under pipeline turbulence conditions and explore the relationship between the convective velocity of turbulent vortices and flow inversion in pipeline turbulence. This study will provide a more reliable theoretical foundation and technical support for DAS-based non-invasive pipeline flow measurement.

## 2. LES of Pipeline Turbulence

**Figure 1:**
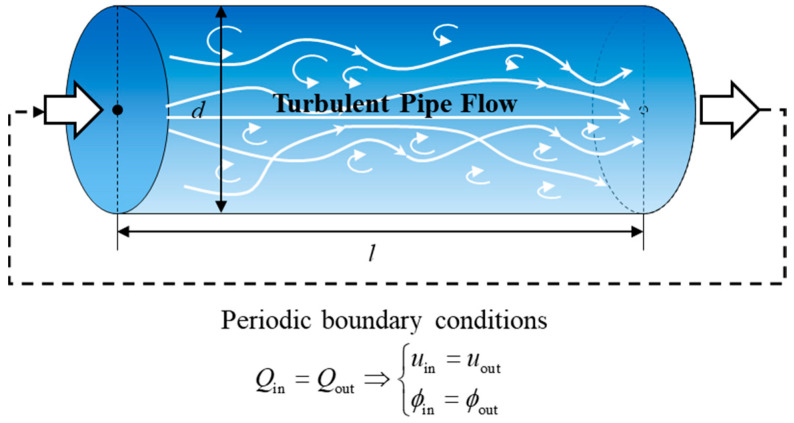
Schematic diagram of the fluid domain model.

The geometric model of the fluid domain for pipeline turbulent flow adopts a standard cylindrical structure. To facilitate comparison with experimental data in subsequent research, the outer diameter of the fluid domain’s geometric model is kept consistent with the inner diameter of the pipeline in the experiment, which is set to d=62 mm. The fluid medium is chosen to be water, and the inlet and outlet flow rates are selected from six typical operating conditions in the experiment. These are converted into mass flow rates of 1.441 kg/s, 2.571 kg/s, 3.007 kg/s, 4.058 kg/s, and 4.571 kg/s, respectively. In determining the axial length of the fluid domain, it is necessary to comprehensively consider the simulation requirements and computational time costs. The numerical simulation of the fluid domain in this paper needs to obtain calculated values under fully developed turbulent flow conditions, while it typically takes a channel development length of 25d~40d for pipe flow to reach the fully developed turbulence state. Traditional inlet boundary condition set up methods significantly increase the size of the computational domain and resource consumption, especially under high Reynolds number conditions, where the computational cost increases exponentially [10]. Therefore, this paper adopts periodic boundary conditions connecting the inlet and outlet, specifying the same total mass flow rate Q at both the inlet and outlet boundaries, i.e., $Q_{in}=Q_{out}$. By forcibly matching the total mass flow rate, velocity field, and SGS properties, this approach achieves self-consistent circulation of the flow field while shortening the computational domain length [11]. The introduction of mathematical continuity constraints by the periodic boundary conditions allows the flow field information at the exit to re-enter the computational domain as the inlet condition, forming a closed physical system. This method effectively reduces the requirement for the length of the computational domain while avoiding the influence of initial disturbances caused by artificially setting inlet conditions on the flow field. Relevant studies show that [12,13], when adopting periodic boundary conditions, taking the length of the computational domain as l=4/3d can achieve numerical solutions meeting accuracy requirements. Early research was constrained by computational resources, so researchers often adopted truncated short-pipe models to control solving scales. With the iterative development of CFD computing technology, current research is no longer constrained by this bottleneck; thus, the length of the fluid domain can be appropriately increased to more comprehensively capture the characteristics of turbulent vortex motion. In this study, after comprehensively weighing the requirements of computational efficiency and result accuracy, the final selected length of the fluid domain is l=0.2 m. This length not only meets the basic requirements of turbulent flow simulation but also ensures that the numerical computation can be completed within a reasonable timeframe. By extending the length of the fluid domain, the spatial evolution process of turbulent vortex structures can be observed more clearly, thereby enhancing the reliability of the simulation. Additionally, a longer fluid domain helps reduce the impact of inlet and outlet boundary conditions on internal flow characteristics, further enhancing the credibility of the results. In summary, the schematic diagram of the fluid domain model is shown in Figure 1, and its structural dimensions and material property parameters are shown in Table 1.

In LES, reasonably determining the grid size is one of the core issues to ensure the accuracy of flow field simulation. In the LES model, a dimensionless length scale parameter, as shown in Equation (1), is usually employed to quantify the grid size [14].

(1) $$\left\{\begin{array}{l} \Delta r^{+}=\Delta r\frac{u_{\tau }}{\nu }\\ \Delta z^{+}=\Delta z\frac{u_{\tau }}{\nu }\\ R\Delta \theta^{+}=R\Delta \theta \frac{u_{\tau }}{\nu } \end{array}\right.$$

**Figure 2:**
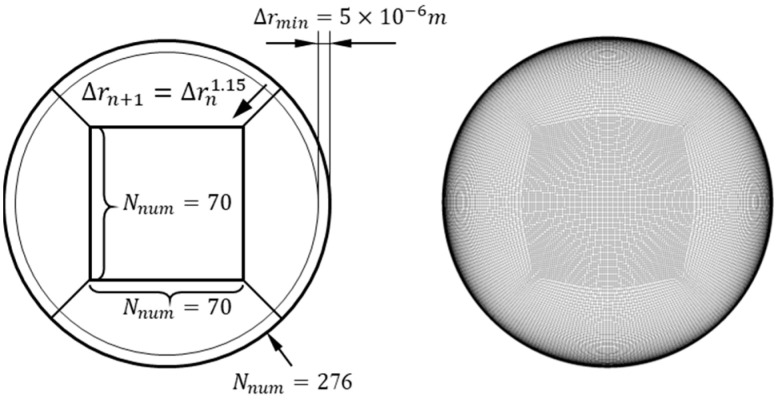
Schematic diagram of fluid domain mesh partitioning.

Here, R represents the radius of the cylindrical fluid domain, where R=d/2; Δr is the radial distance between a specific radial position and the wall of the cylindrical fluid domain; $u_{\tau }$ is the friction velocity; ν is the dynamic viscosity; Δz is the axial grid spacing; RΔθ is the azimuthal grid spacing. The friction velocity can be determined by Equation (2) [15]:

(2) $$u_{\tau }=U\sqrt{\frac{f}{8}}$$

where U is the average fluid velocity, and f is the Darcy friction coefficient, which can be obtained from the classic Moody chart. According to Piomell’s [16] research, the minimum dimensionless values for resolving the boundary layer in the LES model should satisfy the following conditions: $\Delta r^{+}\leq 1,\;\Delta z^{+}\approx 50\sim 100,\;R\Delta \theta^{+}\approx 15\sim 40$. These criteria provide specific guidance for mesh design in numerical simulations. The fluid domain was meshed using the O-grid method. The key characteristic of this approach is that the central region consists of uniform square grids, while the near-wall region gradually transforms to become more radial and perpendicular to the wall, adapting to the physical property variations within the boundary layer. Based on the aforementioned range of dimensionless grid size reference values, the final grid scheme is illustrated in Figure 2. The central square region contains 70 × 70 nodes. The O-grid ring transitioning from the central square region to the cylindrical wall comprises 70 radial nodes, with the outermost radial position set at a distance of $5\times 10^{-6}\;m$ from the boundary. As the radial position approaches the center, the radial grid size increases by a factor of 1.15. The wall interface is divided into 276 circumferential nodes, and the axial direction is divided into 150 nodes. The total number of grids in the fluid domain is approximately 3.59 million. At a flow rate of 4.571 kg/s, the dimensionless grid parameters in the near-wall region are $\Delta r^{+}=0.27$, Δz=63.37, and RΔθ=33.54, all within the reference ranges mentioned above, meeting the requirements for computational grid scales.

During the simulation process, it is first necessary to confirm whether the flow field within the solution domain has reached a fully developed turbulent state, which will serve as the initial condition for numerical simulation. According to turbulence theory, a fully developed turbulent state typically refers to the statistical properties of the flow field remaining unchanged over time and space, satisfying the condition $\partial {\bar{u}}_{i}/\partial t=0$, where ${\bar{u}}_{i}$ denotes the time-averaged velocity component of the flow field. This statistical equilibrium state is an ideal initial condition for fluid numerical simulations because it ensures the reliability and repeatability of subsequent simulation results. The initial condition usually starts from a relatively simple state, allowing the flow to gradually develop through a certain number of time steps until the aforementioned conditions are met and a fully developed turbulent state is achieved. However, due to its high grid resolution, the LES model requires a long time to develop the flow field to a statistically steady state. To shorten the computation time, this paper first uses the RANS-SST model for preliminary calculations before employing the LES model. The RANS-SST model combines the Reynolds-Averaged Navier–Stokes equations (RANS) and the Shear Stress Transport (SST) turbulence model, effectively predicting the average characteristics of turbulence. Its governing equations are shown in Equation (3).

(3) $$\frac{\partial {\bar{u}}_{i}}{\partial t}+{\bar{u}}_{j}\frac{\partial {\bar{u}}_{i}}{\partial x_{j}}=-\frac{1}{\rho }\frac{\partial \bar{p}}{\partial x_{i}}+\frac{\partial }{\partial x_{j}}\left[\left(\nu +\nu_{t}\right)\frac{\partial {\bar{u}}_{i}}{\partial x_{j}}\right]$$

where $\nu_{t}$ is the turbulent viscosity coefficient calculated by the SST model. In the RANS-SST model, as the number of time steps increases, the statistical properties of the flow field gradually reach a steady state, satisfying $\partial {\bar{u}}_{i}/\partial t\approx 0$. Subsequently, this stable solution serves as the initial condition for the LES-Smagorinsky model.

Additionally, in setting the initial conditions for the LES model, the Courant number is a key dimensionless parameter used to measure the relative relationship between the time step and spatial step. It is defined by Equation (4).

(4) $$C=\bar{u}\frac{\Delta t}{\Delta z}$$

where $\bar{u}$ is the average flow velocity, Δt is the time step, and Δz is the grid size in the flow direction. This parameter physically represents the number of grid cells that a fluid element migrates within one time step, directly reflecting the match between temporal and spatial resolutions in numerical computations. In explicit time advancement schemes, the choice of the Courant number critically affects computational stability. If the Courant number is too large, it may lead to divergence of the numerical solution; conversely, while a smaller Courant number helps improve stability, it significantly increases computational cost. Through systematic numerical verification, it was determined that a time step of Δt=0.0005 s ensures that the Courant number remains less than 1 under all conditions, thereby avoiding numerical instability caused by excessively large time steps. Furthermore, during actual calculations, strict monitoring of residual convergence criteria confirms that each time step reaches the set convergence standard after at most five iterations.

**Figure 3:**
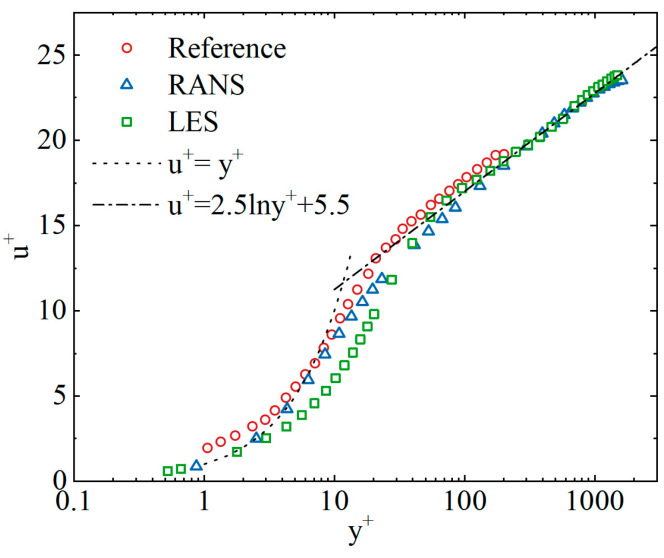
Normalized velocity profile [17].

To verify the accuracy of the LES solver in predicting the research problem addressed in this paper, the statistical results from the LES solution can be compared with the wall logarithmic law and experimental research results presented in the text. The wall motion law is expressed as the relationship between the dimensionless fluid velocity and the dimensionless wall distance $y^{+}$, where $u^{+}=U/u_{\tau }$ and $y^{+}=yu_{T}/v$. For a cylindrical tube, $u^{+}$ and $y^{+}$ follow a linear relationship when 0 < $y^{+}$ < 5 and conform to $u^{+}=2.5\ln y^{+}+5.5$ when $y^{+}>30$. Figure 3 plots the results calculated using the steady-state RANS model and the LES model at a flow rate of 4.571 kg/s as well as the experimental results from reference [17]. It can be seen that the time-averaged statistics from the LES roughly follow the wall logarithmic law, indicating that the computational model in this paper has a certain degree of accuracy.

## 3. Verification of the Taylor Frozen Hypothesis in LES

### 3.1. Temporal and Spatial Evolution of Turbulent Kinetic Energy Field in Pipelines

**Figure 4:**
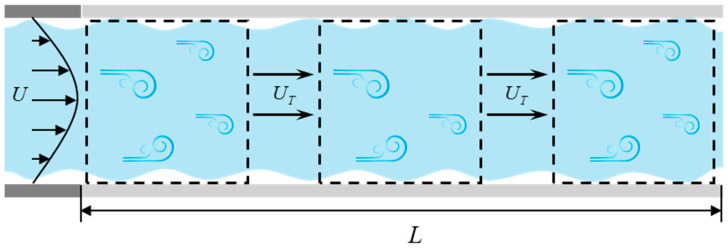
Diagram of the Taylor freezing hypothesis principle.

The Taylor freeze hypothesis suggests that in a turbulent field, when the average velocity of the fluid is much greater than the turbulent fluctuation velocity, the turbulent vortex structures can be approximately regarded as being in a “frozen” state [18]. The key to this hypothesis is that the spatial distribution and morphology of the turbulent vortices will not significantly change due to local fluctuations over a short period of time. Instead, they are carried through the flow field space at a relatively stable convection velocity. A schematic diagram illustrating this principle is shown in Figure 4.

Through time-series analysis of the turbulent kinetic energy distribution, the dynamic change patterns of vortices along the temporal dimension can be captured [19]. A yOz plane cross-section of the model is selected, and the instantaneous velocity field data (u,v,w) for all nodes on this cross-section are extracted from the LES results. The instantaneous turbulent kinetic energy E(k) in the chosen cross-sectional turbulence field can be quantitatively characterized using Equation (5).

(5) $$E(k)=\frac{1}{2}({u^{\prime }}^{2}+{v^{\prime }}^{2}+{w^{\prime }}^{2})$$

**Figure 5:**
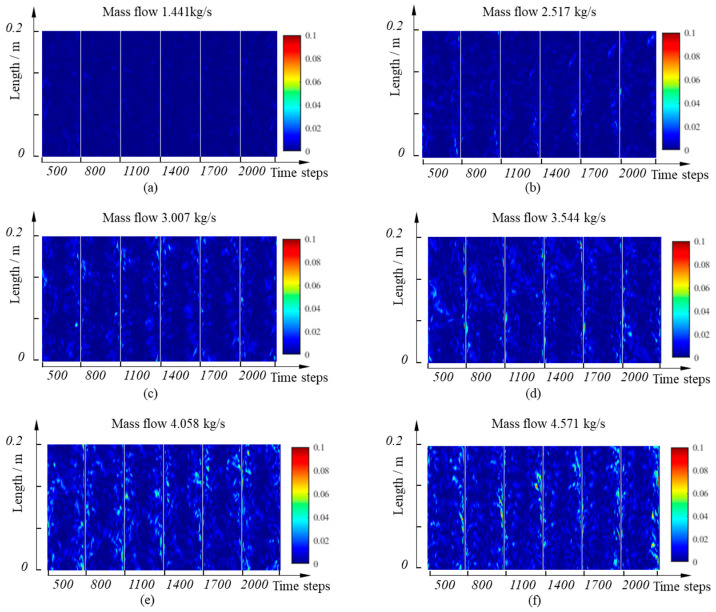
Spatial and temporal distribution characteristics of the turbulent kinetic energy field in the pipeline under different flow conditions. (**a**) Flow rate 1.441 kg/s; (**b**) Flow rate 2.517 kg/s; (**c**) Flow rate 3.007 kg/s; (**d**) Flow rate 3.544 kg/s; (**e**) Flow rate 4.058 kg/s; (**f**) Flow rate 4.571 kg/s.

The results of six mass flow rate simulations are processed using the method described above. To more intuitively observe the intensity variation in turbulent vortices and their spatial migration patterns, the turbulent kinetic energy distributions at the 500th, 800th, 1100th, 1400th, 1700th, and 2000th time steps are extracted and horizontally concatenated. The results are shown in Figure 5.

By comparing the distribution of turbulent kinetic energy fields at different time steps under the same flow rate, the dynamic characteristics of turbulent vortices moving along the flow direction over time can be observed. This phenomenon indicates that the movement of turbulent vortices has significant directionality and time dependence, with their movement speed increasing as the average flow rate rises. This result aligns with Taylor’s frozen hypothesis: in fully developed turbulence, vortex structures are carried and propagated by the main fluid, with propagation speeds close to the average velocity of the fluid. The distribution of turbulent kinetic energy fields shows notable differences under various flow conditions. When the flow rate is 1.441 kg/s, the turbulent kinetic energy values are relatively low, primarily represented by blue to light-blue blocks, indicating limited generation of turbulent vortices and a more stable flow state. As the flow rate increases to 2.517 kg/s and 3.007 kg/s, the proportion of light-yellow and yellow blocks rises, signifying an increase in turbulence intensity. With the flow further increasing to 3.544 kg/s, 4.058 kg/s, and 4.571 kg/s, the number of yellow and red blocks increases, showing that high-intensity turbulent areas expand from near the wall towards the center, demonstrating continuously enhanced and uneven turbulence intensity. This expansion may relate to changes in the boundary layer and increased interaction between the mainstream and boundary layer, reflecting the rising complexity of turbulent structures. In summary, the movement of turbulent vortices along the flow direction accords with Taylor’s frozen hypothesis, with their movement speed positively correlated with the average flow velocity. High-turbulence areas mainly lie near the wall. However, as the flow rate increases, the vortex intensity significantly strengthens, structural complexity rises, and high-intensity areas gradually extend towards the pipe center.

### 3.2. Calculation of Turbulent Vortex Convection Velocity Based on LES Data

In non-invasive flow measurement based on Taylor’s frozen hypothesis, a sensor array is laid on the outer wall of the pipe to measure the spatiotemporal signals of pressure fluctuations caused by turbulent vortices. A pipeline flow non-invasive measurement system based on DAS (distributed acoustic sensing) achieves this array sensing through optical fibers uniformly wound around the outer wall of the pipe. The convection velocity $U_{T}$ of pressure fluctuations related to turbulent vortices can be calculated using the formula $U_{T}=f/k$, where f represents the time frequency of turbulent vortex pressure pulsations, and k represents the wavenumber of turbulent vortex pressure pulsations in the axial direction of the pipe. In the numerical simulation settings of this paper, the grid in the boundary area consists of 276 circumferential nodes and 150 axial nodes forming a spatial discretization system, with each node containing pressure fluctuation time-history data for 2000 time steps. To simulate the measurement signals of the DAS system, the pressure fluctuation data of all nodes in each circumferential section are summed and averaged, ultimately forming a 150 × 2000 pressure fluctuation spatiotemporal matrix. A two-dimensional Fourier transform is applied to this matrix to convert the spatiotemporal domain signal into the frequency–wavenumber domain, thereby constructing an FK spectrum. The mathematical expression of the two-dimensional Fourier transform is shown in Equation (6).

(6) $$S\left(k,f\right)=\frac{1}{MN}\sum_{m=0}^{M-1}\sum_{n=0}^{N-1}p\left(x_{m},t_{n}\right)e^{-i2\pi \left(\frac{ft_{n}}{N}+\frac{kx_{m}}{M}\right)}$$

**Figure 6:**
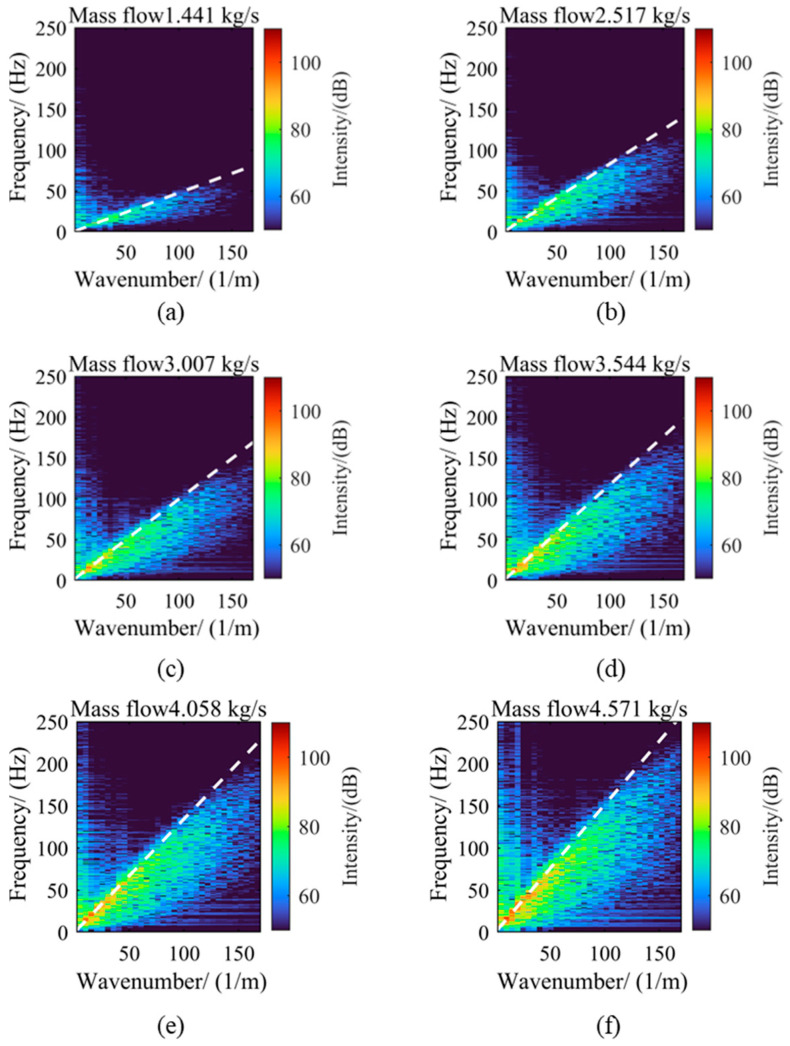
Wall pressure fluctuation FK spectrum under different flow conditions. (**a**) Flow rate 1.441 kg/s; (**b**) Flow rate 2.517 kg/s; (**c**) Flow rate 3.007 kg/s; (**d**) Flow rate 3.544 kg/s; (**e**) Flow rate 4.058 kg/s; (**f**) Flow rate 4.571 kg/s.

Figure 6 shows the FK spectra under six different flow conditions. To enhance visualization contrast, a logarithmic compression method was used to process the amplitude data after the two-dimensional Fourier transform during data processing. This method effectively suppresses the over-saturation of strong signals while preserving low-amplitude details through a nonlinear mapping formula, thus improving the recognizability of weak-signal areas in the spectrum. To further analyze the relationship between the energy distribution of the FK spectrum and the average flow velocity, a dashed line passing through the origin was drawn in each spectrum, with its slope corresponding to the average flow velocity under each condition. By comparing the FK spectra under different flow conditions, it can be found that as the flow increases, the energy distribution pattern changes significantly. When the flow rate is 1.441 kg/s, the energy in the FK spectrum is smaller and mainly concentrated in the lower wavenumber and frequency regions, manifested as bright distributions in the lower-left corner area. As the flow gradually increases to 4.571 kg/s, the energy gradually increases, and the energy distribution gradually shifts towards higher wavenumbers and frequencies. Notably, for each flow condition, the reference line representing the average flow velocity basically passes through the area of maximum energy in the FK spectrum, indicating a strong correlation between the average flow velocity and the main energy distribution. Moreover, regardless of how the flow changes, the high-energy areas in the FK spectrum always distribute in the lower-left corner, i.e., the lower wavenumber and frequency regions. This phenomenon shows that in pipeline turbulence, large-scale vortices of medium and low frequencies play a dominant role. Low wavenumbers indicate larger spatial scales of vortices, while low frequencies suggest slower temporal evolution, which can maintain their dynamic characteristics for a longer period. This conclusion is consistent with the phenomena described by Taylor’s frozen hypothesis.

**Figure 7:**
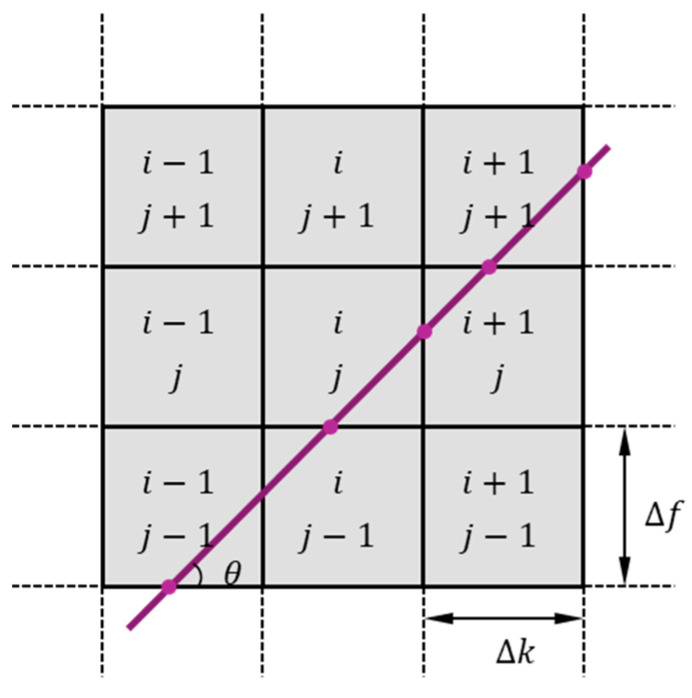
Schematic diagram of the core idea of line recognition algorithm.

To further quantify the relationship between the average flow velocity and the convection velocity, it is necessary to extract the oblique lines representing the convection velocity in the FK spectrum diagram and calculate their slopes. This paper proposes an efficient recognition algorithm suitable for frequency–wavenumber space analysis by integrating the concept of Radon transform. The schematic diagram of the algorithm is shown in Figure 7. Through the integral method represented by Equation (7), this algorithm establishes a mapping relationship between the straight lines passing through the origin of the spectrogram in different directions and the data in the FK spectrogram space.

(7) $$I_{k}=\frac{1}{L_{k}}\sum_{(i,j)\in P_{k}}l_{ijk}F_{ij}$$

In this equation, $P_{k}$ denotes the set of grid cells penetrated by the k-th slope line, $L_{k}=\sum l_{ijk}$ represents the total path length, and $F_{ij}$ stands for the Fourier modulus of the FK spectrogram unit (i,j).

**Figure 8:**
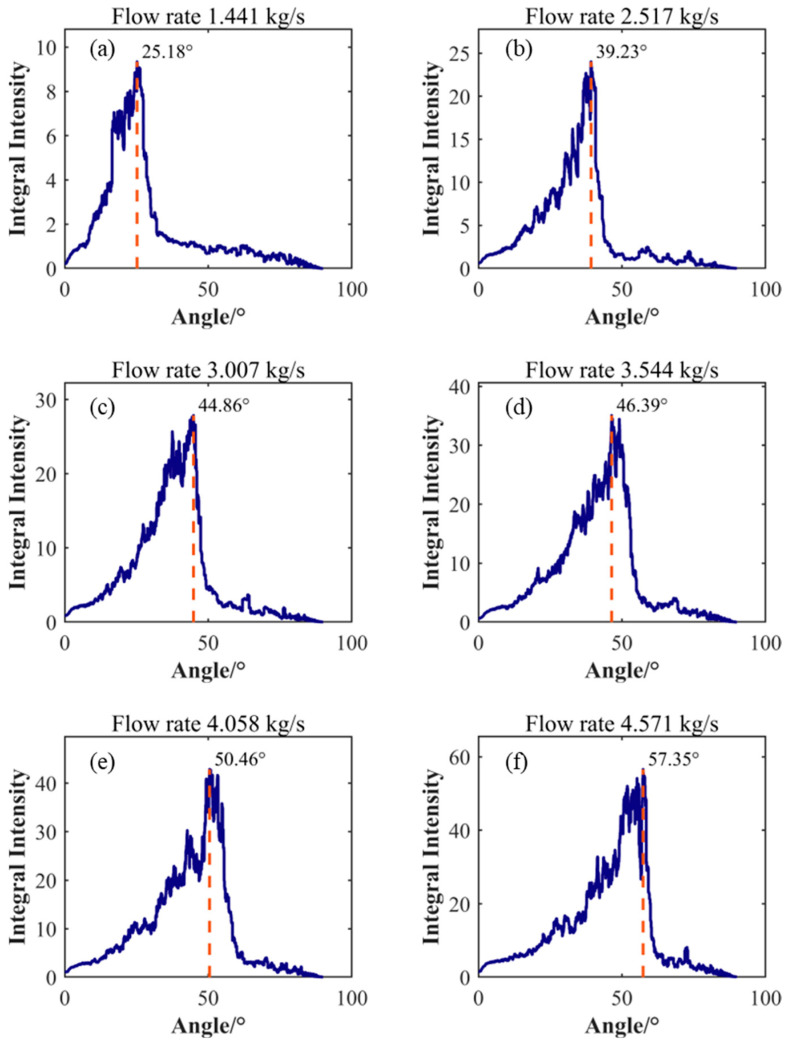
Identification of characteristic slope angles under different flow conditions. (**a**) Flow rate 1.441 kg/s; (**b**) Flow rate 2.517 kg/s; (**c**) Flow rate 3.007 kg/s; (**d**) Flow rate 3.544 kg/s; (**e**) Flow rate 4.058 kg/s; (**f**) Flow rate 4.571 kg/s.

Figure 8 shows the results of analyzing the energy distribution in the FK spectrogram using a line detection algorithm. The horizontal axis in the figure represents the angle of the line, while the vertical axis represents the corresponding integrated intensity. By analyzing the integrated intensity, the angle corresponding to the maximum integrated intensity can be determined. This angle is defined as the tilt angle of the characteristic diagonal line representing the primary turbulent vortex convection velocity. To quantify the relationship between the convection velocity and the average velocity, the convection velocity represented by the slope was calculated by extracting the characteristic angle. The average flow velocity was obtained by averaging the axial instantaneous velocities of all nodes in the LES calculation results and employing statistical methods. The relative offset between the convection velocity and the average velocity is shown in Table 2. The data in the table indicates that the convection velocity of the vortex increases with increasing flow rate. In low flow conditions ranging from 1.441 kg/s to 3.007 kg/s, the difference between the convection velocity and the average velocity is small, showing a negative offset with a maximum offset of −2.07%. In moderate flow conditions at 3.544 kg/s and 4.058 kg/s, the offset between the convection velocity and the average flow velocity increases, reaching a maximum deviation of −10.64%, which is significantly lower than the average velocity. However, under high flow conditions at 4.571 kg/s, the convection velocity exceeds the average velocity, with an offset value of 3.08%.

The comparison between the convection velocity and the average flow velocity results reveals a nonlinear characteristic in their offsets, particularly under certain high flow conditions where significant deviations exist between the calculated convection velocity and the average flow velocity. This phenomenon can be attributed to the complex nature of pipe turbulence, which differs from planar flow fields. In cylindrical pipe flow, due to the presence of axial symmetry and circumferential flow patterns, non-primary vortices influence pressure fluctuations of primary vortices—a fact reflected in Figure 8. As flow increases, multiple secondary peaks with similar intensities appear near the main peak. Clearly, the conclusion derived from planar flow fields, that identifying the line with maximum integrated energy in the FK spectrum as the vortex convection velocity method, is not applicable here. Further investigation is needed into whether the energy distribution in specific directions is correlated.

### 3.3. Method for Enhancing Directional Features of FK Spectrogram Based on Cross-Correlation

In response to the above problem, this paper designs a method for enhancing the dispersion characteristics of FK spectrum based on the cross-correlation method, improving the recognition of direction-related energy in the FK spectrum. The energy distribution of the dispersion signal in the FK spectrum exhibits local linear correlation characteristics. The observed spectrum is shown in Equation (8):

(8) S(f,k)=D(f,k)+N(f,k)

where D(f,k) is the dispersion signal component that satisfies the dispersion relation f=k⋅U, and N(f,k) is the noise component of non-dispersive energy, with isotropic energy distribution characteristics. To quantify the correlation between energy and direction, a direction-sensitive elliptical window model is constructed. A rotatable elliptical dispersion template $T_{\theta }(x,y)$ is defined in Equation (9):

(9) $$T_{\theta }(x,y)=\left\{\begin{array}{ll} e^{-\frac{{\left(x^{\prime }\right)}^{2}}{2\sigma_{x}^{2}}-\frac{{\left(y^{\prime }\right)}^{2}}{2\sigma_{y}^{2}}}, & \mathrm{if}\;\frac{{\left(x^{\prime }\right)}^{2}}{a^{2}}+\frac{{\left(y^{\prime }\right)}^{2}}{b^{2}}\leq 1\\ 0, & \end{array}\right.$$

where $\sigma_{x},\;\sigma_{y}$ are Gaussian attenuation parameters of the major and minor axes, and a, b are the semi-major and semi-minor axes of the ellipse, respectively. To ensure that the ellipse covers 99% of the Gaussian energy, set $a=3\sigma_{x}\;,b=3\sigma_{y}$. Using an elliptical template with an adjustable aspect ratio can naturally adapt to the directional characteristics of energy distribution in coherent structures, thereby more accurately identifying their main dispersion direction. In addition, by rotating the ellipse, all possible convection directions and velocities can be scanned. The major and minor semi-axes of the ellipse are defined as 3σ to minimize the influence of noise and weak signals in the edge regions of the template.

The conversion from the local window coordinate system (p,q) to the rotated coordinate system $\left(x^{\prime },y^{\prime }\right)$ is carried out using the formula in Equation (10):

(10) $$\left\{\begin{array}{l} x^{\prime }=p\cdot \cos\theta -q\cdot \sin\theta \\ y^{\prime }=p\cdot \sin\theta +q\cdot \cos\theta \end{array}\right.$$

where θ is the angle of the major axis direction of the direction-sensitive elliptical window model. Since it is not possible to directly determine the unknown dispersion lines in the FK spectrum, multi-directional elliptical dispersion templates $T_{\theta }$ are directly constructed, where $\theta \in [0^{{}^{\circ}},1^{{}^{\circ}},2^{{}^{\circ}},3^{{}^{\circ}},\ldots ,180{}^{\circ}]$.

For each position $\left(f_{0},k_{0}\right)$ in the spectrum, match the local window $W(f_{0},k_{0})$ with each directional template $T_{\theta }$, calculating the Normalized Cross-Correlation coefficient (NCC) as shown in Equation (11).

(11) $$NCC(f_{0},k_{0};\theta )=\frac{\sum_{(i,j)\in W}[S_{f_{0}+i,k_{0}+j}-\mu_{W}]\cdot [T_{\theta }(i,j)-\mu_{T}]}{\sqrt{\sum {\left(S_{f_{0}+i,k_{0}+j}-\mu_{W}\right)}^{2}\cdot \sqrt{\sum {\left(T_{\theta }(i,j)-\mu_{T}\right)}^{2}}}}$$

where W is a sliding window centered at $\left(f_{0},k_{0}\right)$, $S_{f,k}$ is the energy value of the FK spectrum at frequency f and wavenumber k, $\mu_{W}$ is the mean of the local sliding window, and $\mu_{T}$ is the mean of the corresponding directional template. By calculating the normalized cross-correlation coefficients for every position in the entire FK map with all directional templates, the normalized cross-correlation coefficients for all positions in the FK spectrum can be obtained.

Generate a confidence map C(f,k) as shown in Equation (12).

(12) $$C(f,k)=\max_{\theta_{i}}NCC(f,k;\theta_{i})$$

Here, C(f,k) quantifies the likelihood that the current point belongs to a dispersive linear feature. When confidence is high (C→1), it indicates that the sliding window at the current position $\left(f_{0},k_{0}\right)$ has strong directional continuity energy, indicating strong dispersion relations. When the confidence is low (C→0), it represents isotropic noise or isolated anomalies at the current position $\left(f_{0},k_{0}\right)$, proving weak or non-existent dispersion relations.

A non-parametric quantile threshold method is used here to avoid the sensitivity of fixed thresholds to data distribution. The corresponding threshold formula is shown in Equation (13).

(13) $$\mathrm{Threshold}=Q_{\alpha }(\{C(f,k)\}),\;\alpha =90\%$$

where $Q_{\alpha }$ represents the α% quantile of the confidence distribution. Finally, use this threshold to binarize the confidence map, resulting in the segmented confidence map as shown in Equation (14).

(14) $$M_{\mathrm{initial}}(f,k)=\left\{\begin{array}{ll} 1, & \mathrm{Confidence}(f,k)>\mathrm{Threshold}\\ 0, & \mathrm{otherwise} \end{array}\right.$$

Fill small holes and connect broken dispersion lines in the threshold-segmented confidence map *Mask*(*f*,*k*) using morphological closing operations as shown in Equation (15).

(15) $$M_{closed}=\mathrm{Dilate}(\mathrm{Erode}(M_{initial},SE),SE)$$

where Dilate is the morphological dilation operation, Erode is the morphological erosion operation, and SE is the structural element kernel in morphology.

Finally, to avoid the influence of noise, small noisy blocks are filtered out to obtain the morphologically optimized confidence map, as shown in Equation (16).

(16) $$M_{final}=M_{closed}\backslash \{Area(R_{i})<50\}$$

where $\left\{Area(R_{i})<50\right\}$ denotes the operation of eliminating noisy blocks with area less than 50 pixels. Through this operation, the extraction effect of the dispersion features is further enhanced.

**Figure 9:**
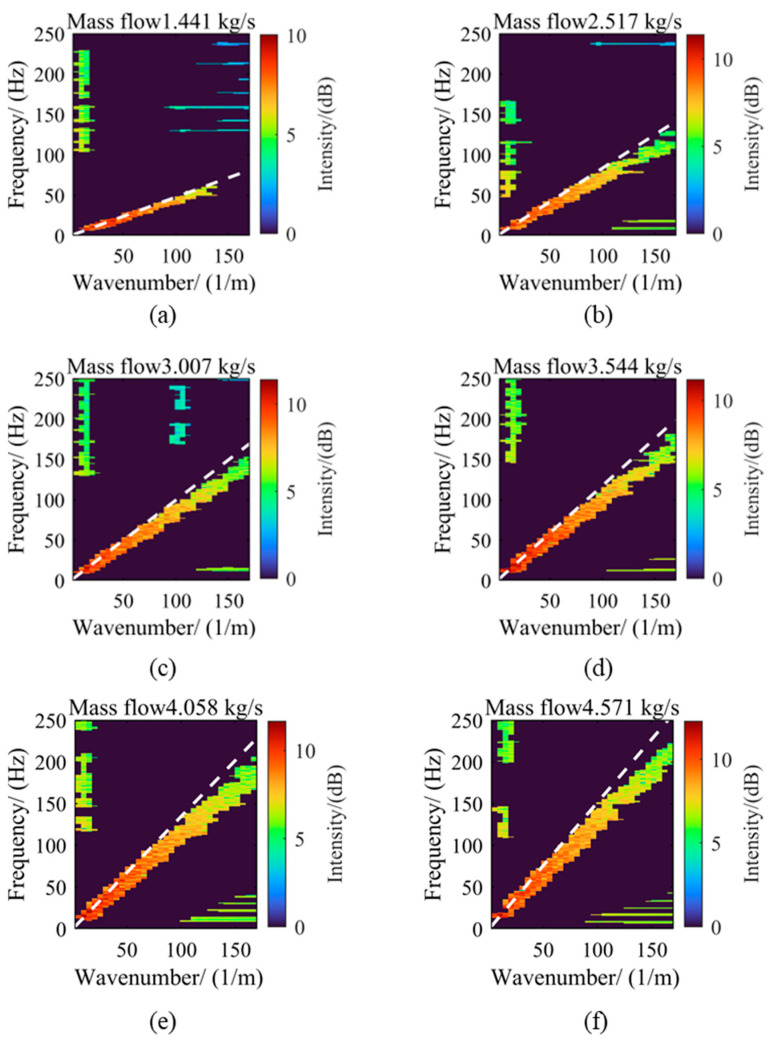
Directional feature enhanced FK spectrum. (**a**) Flow rate 1.441 kg/s; (**b**) Flow rate 2.517 kg/s; (**c**) Flow rate 3.007 kg/s; (**d**) Flow rate 3.544 kg/s; (**e**) Flow rate 4.058 kg/s; (**f**) Flow rate 4.571 kg/s.

Based on the above algorithm, FK spectra under six flow conditions were processed, with results shown in Figure 9. The white dotted line in the figure indicates the average velocity under the corresponding flow condition. From an overall trend analysis, as the flow increases, the principal diagonal feature of the energy distribution becomes more significant and continuous, and the energy intensity significantly enhances along this direction, aligning better with the white dotted line. This indicates that the direction feature enhancement method effectively captures and highlights the dominant direction features of fluid motion under different flow conditions.

**Figure 10:**
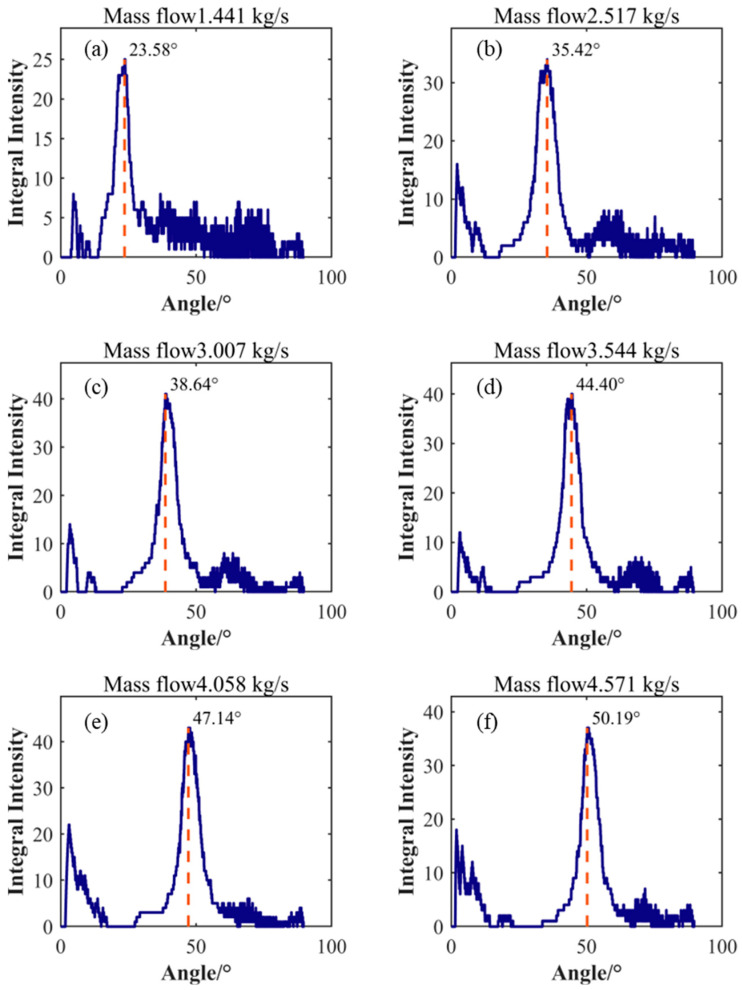
Enhancement of Direction Features for Identifying Slope Angles of Characteristics in Different Flow Conditions (**a**) Flow rate 1.441 kg/s; (**b**) Flow rate 2.517 kg/s; (**c**) Flow rate 3.007 kg/s; (**d**) Flow rate 3.544 kg/s; (**e**) Flow rate 4.058 kg/s; (**f**) Flow rate 4.571 kg/s.

After direction feature enhancement processing, feature line identification of the enhanced FK spectrum was conducted, with results shown in Figure 10. Comparing Figure 8 with Figure 10, it can be observed that after direction feature enhancement processing, the integral intensity distribution with angular variation exhibits a significant unimodal characteristic. Energy is primarily concentrated in the main peak region, while the energy distribution on both sides of the peak is significantly reduced and much lower than the peak level. This phenomenon indicates that the direction feature enhancement effectively suppresses interference components in non-target directions, thus improving the concentration and distinguishability of the signal.

**Figure 11:**
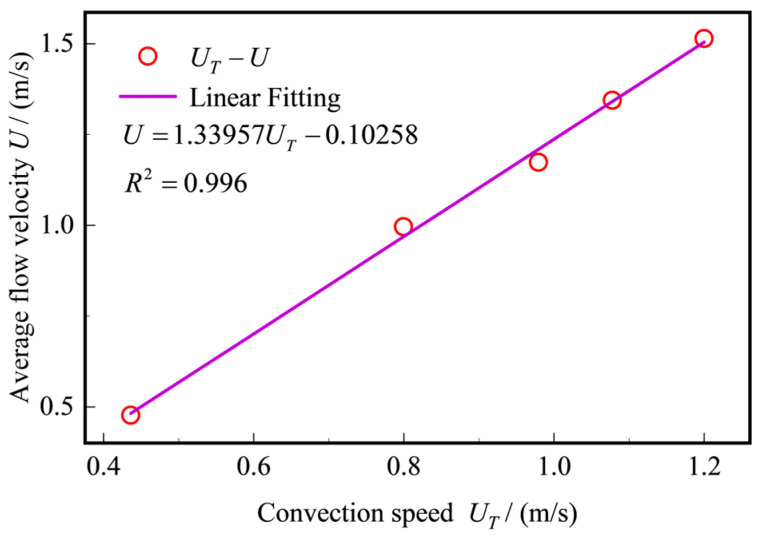
Fitting relationship graph between convection velocity and average velocity.

Figure 11 shows the fitting relationship between the convective velocity and the average flow velocity. The fitting equation is $U=1.33957U_{T}-0.10258$, with a linear fit of $R^{2}=0.996$, indicating that the fitting results have a high linear correlation. Table 3 lists the specific calculation results of the average flow velocity, convective velocity, calculated average flow velocity, and relative error. Through the analysis of the data, it can be seen that the relative error between the calculated average flow velocity and the standard average flow velocity is controlled within 3%. This result further verifies the feasibility of the method based on the Taylor frozen hypothesis principle in pipeline flow measurement.

## 4. Experiments and Discussion

**Figure 12:**
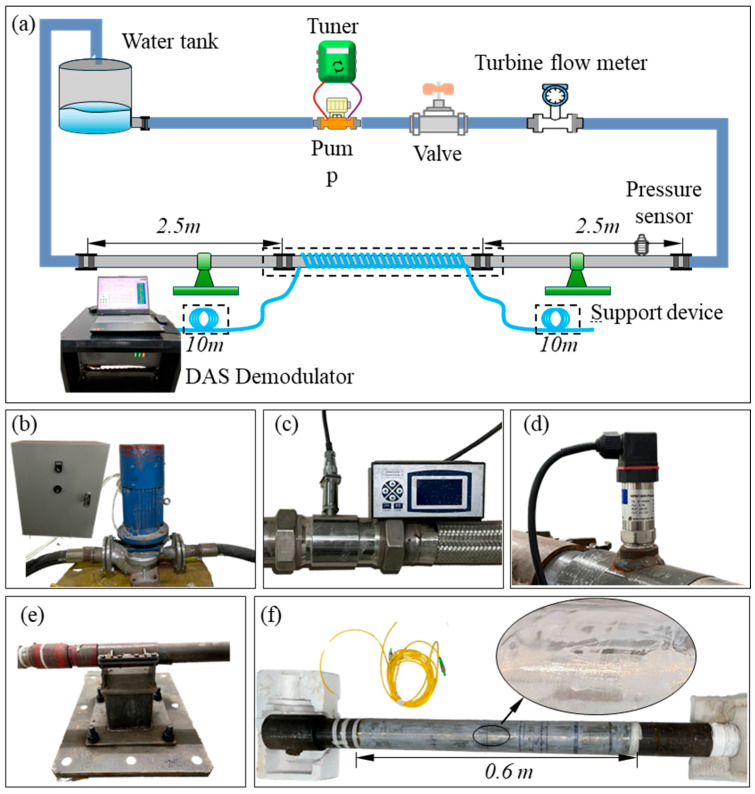
Experimental setup illustrated with equipment diagram (**a**) Schematic diagram of the experimental system for measuring flow in a circular pipeline. (**b**) Centrifugal pump and variable frequency drive. (**c**) Turbine flow meter. (**d**) Dynamic pressure sensor. (**e**) Pipeline support device. (**f**) Test pipe section.

To validate the pipeline flow measurement method based on the Taylor Frozen Hypothesis, a circulating pipeline flow measurement experimental system, as shown in Figure 12a, was constructed. The core components of this system include a water tank, a centrifugal pump, inlet and return hoses, a test pipe, and supporting devices. Water, serving as the fluid medium in the experiment, is stored in a water tank with a volume of 100 L. The water tank is connected to the centrifugal pump via an inlet pipe, and the centrifugal pump pumps water into the loop pipe, creating a stable flow state during the process. Following the loop pipe, there is a test pipe with an outer diameter of 73 mm and an inner diameter of 62 mm. A 2.5 m length of fluid development pipe is connected to the front section of the test pipe, primarily serving to ensure that the fluid reaches a fully developed turbulent state before entering the test section, thus meeting the stringent requirements for flow conditions in the experiment. Within the piping circuit, a high-precision flowmeter and dynamic pressure sensors are installed. The flowmeter selected is the LWGY-50 type turbine flowmeter, with a range of 10 to 300 L/min and an accuracy level of 0.5, used for real-time measurement of the pipeline system’s flow rate as a calibration value. The pressure sensor selected is the HPM180H type dynamic pressure sensor, with a range of 0 to 50 kPa, a response frequency of 2 kHz, and a measurement accuracy of 0.1% F.S., used to determine whether the pipeline flow rate has reached a fully developed state. The DAS demodulation equipment used in the experiment was manufactured by AP Sensing, with the model number N52-R50. The maximum sampling frequency of this device can reach 20,000 Hz (the maximum sampling rate was selected in the experiment). The spatial sampling interval along the fiber length was set to 1.25 m, and the system’s spatial resolution was 5 m.

To capture the dynamic strain variations associated with fluid pressure fluctuations, approximately 600 m of optical fiber are tightly wound around the test pipe, covering an axial length of approximately 0.6 m. This winding configuration ensures that the fiber effectively measures circumferential strain on the pipe cross-section. According to principles of structural mechanics, internal fluid pressure induces circumferential strain on the pipe wall, which is linearly related to the pressure under the assumption of small deformations and constant material properties. The distributed acoustic sensing (DAS) system detects axial strain along the fiber direction; therefore, by winding the fiber circumferentially around the pipe, the system can indirectly capture pressure-induced strain at a given cross-section. In addition, the fiber winding length of 600 m ensures sufficient spatial sampling along the axial direction of the pipe. Given that the DAS system has a spatial resolution of 5 m, measurement points are sampled at intervals of 5 m to prevent signal aliasing. The selected winding length allows for multiple independent measurement points, thereby increasing data reliability and spatial coverage. Finally, the fiber is coated with a thin and uniform layer of AB glue to enhance mechanical coupling between the fiber and the pipe surface, ensuring that the fiber accurately reflects the deformation of the pipe wall.

Before conducting the experiment, the optical fiber was connected to the DAS demodulation device, and the calibration of the positions at both ends of the fiber wound around the test pipe section was determined by tapping the pipeline. To eliminate the influence of the rotational frequency of the centrifugal pump under different flow conditions on the fluid flow characteristics, the output frequency of the inverter was fixed at 50 Hz during the experiment, and precise flow control was achieved by adjusting the valve opening. The length of the pipeline between the valve and the test pipeline was set to 8 m to ensure that the fluid had sufficient development distance before entering the test section. After each adjustment of the flow rate, the signal from the dynamic pressure sensor was extracted and its average value was calculated. Subsequent operations were carried out after the average pressure stabilized. Once the flow field stability was confirmed, the volume flow rate measured by the flow meter was recorded, and relevant data was simultaneously obtained from the DAS system. A total of six sets of volume flow data were recorded in the experiment: 274.26 L/min, 243.48 L/min, 212.64 L/min, 180.42 L/min, 151.02 L/min, and 86.46 L/min (corresponding mass flow rates were the same as those in the simulation settings). The sampling rate of the DAS system was 20,000 Hz, and the sampling time was 1 s.

The spatiotemporal data collected by the DAS system under each flow condition were expressed in the form of an [m,n] matrix, where m=495 corresponds to the number of measurement points generated by the fiber wound around the test pipe section, with the fiber length between adjacent measurement points being 1.25 m; n= 200,000 represents the total number of time samples. Since the spatial resolution of the DAS system is 5 m, to avoid signal crosstalk between measurement points, an average value was calculated for every four columns of the original data, thereby reducing the number of measurement points to 123. Further, by selecting points at intervals, 62 effective measurement points were finally retained, with an interval distance of 5.6 mm along the axial direction of the pipe. According to the results of LES, under maximum flow conditions, the frequency of pressure fluctuations caused by the main vortices in the turbulent field was below 250 Hz. To reduce data redundancy and improve processing efficiency, the original data were down-sampled in the time dimension, with the sampling rate set to 2000. After the above processing, the data for each flow rate were converted into a 62 × 2000 matrix.

During the measurement process, the DAS system captures a large amount of environmental and system noise, which can mask useful signals and hinder result analysis. Therefore, it is necessary to first denoise the DAS measurement data. Effective signals affected by noise interference often exhibit an increasing standard deviation trend. First, the size of the standard deviation of the data within the comparison window is compared to the overall standard deviation of the signal. When the standard deviation of the data within the window is greater than the overall, a certain filtering window W is selected with each point of the measurement signal as the center, removing the maximum and minimum values within the window. The remaining elements are collectively labeled as H. The weights are calculated by first finding the Mean(H) of the elements in H, then using Equation (17) to calculate the corresponding weights of the elements in H, and normalizing them.

(17) $$u_{i}^{\prime }=\frac{1/(1+Max(D_{k},T))}{\sum_{k=1}^{L}1/(1+Max(D_{k},T))}$$

The weight of each point in the element collection H within the window is $u^{\prime }$, with the number of elements being L, consistent with the window value. The absolute difference between the values of elements in set H and the mean value Mean(H) is $D_{k}$, as shown in Equation (18). T is calculated in Equation (19) as the average value of all $D_{k}$, representing a threshold. The algorithm uses a threshold optimization principle when calculating the weights of each point: if the absolute difference $D_{k}$ between a point in H and its internal mean value is greater than the threshold *T*, the weight is determined by $D_{k}$. If $D_{k}$ is less than the threshold *T*, the weight is determined by *T*.

(18) $$D_{K}=(H_{K}-Mean(H[f(i)]))$$

(19) $$T=\frac{\sum_{k=1}^{L}\left|H_{K}-Mean(H[f(i)]\right)|}{L}$$

Further, all the element points in the set H are weighted with their corresponding weights, and the result is used as the output of the center point of the filter window W, as shown in Equation (20).

(20) $$f{\left(i\right)}^{\prime }=\sum_{k=1}^{L}H_{K}(i)\times u_{i}^{\prime }S=P\times B$$

**Figure 13:**
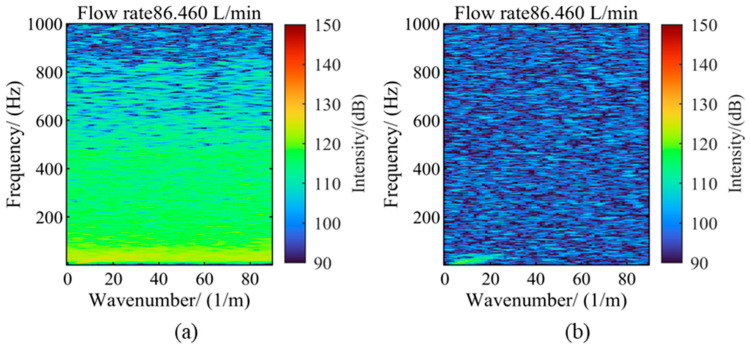
Measured data FK spectrum (**a**) before denoising (**b**) after denoising.

Figure 13 shows the FK spectrum comparison before and after denoising at a flow rate of 86.46 L/min. It can be seen from the figure that after denoising, the characteristic lines are clearly visible in the FK transform of the data, indicating that the above method has a good denoising effect.

**Figure 14:**
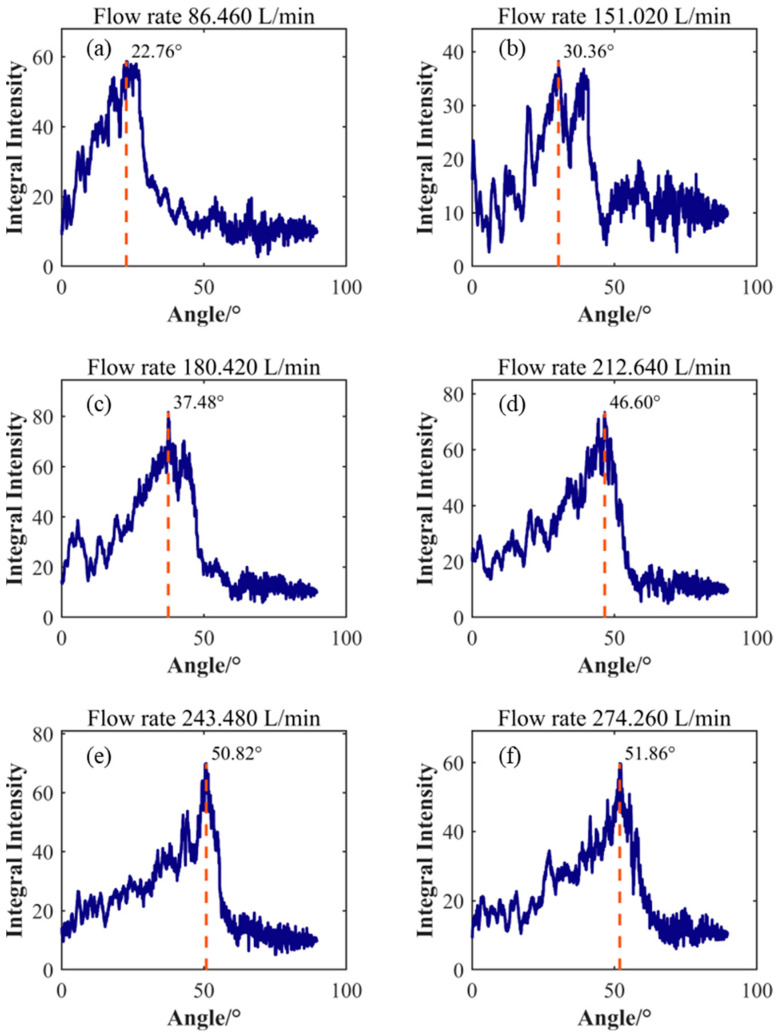
Identification of characteristic diagonal angles under different flow conditions in experimental testing. (**a**) Flow rate 86.460 L/min; (**b**) Flow rate 151.020 L/min; (**c**) Flow rate 180.420 L/min; (**d**) Flow rate 212.640 L/min; (**e**) Flow rate 243.480 L/min; (**f**) Flow rate 274.260 L/min.

After denoising the data, directional feature enhancement processing was performed, followed by the identification of characteristic straight lines. The results are shown in Figure 14. Table 4 compares the measurement data from a standard flowmeter, flow data calculated based on the Taylor frozen hypothesis, and flow data calculated based on the fluid-induced vibration (FIV) principle. The experimental data in the table indicate that the flow calculation method based on the Taylor frozen hypothesis demonstrates systematic advantages in terms of measurement accuracy. As the standard flowmeter reading gradually increased from 86.46 L/min to 274.26 L/min, the relative error of the Taylor frozen hypothesis calculation showed a decreasing trend, with the error margin dropping continuously from an initial 19.63% to 3.87%, indicating that this method has greater stability in high-flow ranges. In contrast, the relative error range of the FIV calculation is larger, with the maximum error of 18.94% occurring at the 180.42 L/min measurement point. Moreover, the calculated flow rate was smaller than the previous one. Although the error change began to stabilize after the flow exceeded 243.48 L/min, no significant convergence characteristics were observed. From the perspective of absolute error distribution, the standard deviation of the Taylor frozen hypothesis error (±4.83%) is lower than that of the FIV method error (±6.95%), indicating greater reliability in measurement repeatability for the former.

## 5. Discussion

The present research addresses an important gap in the application of Taylor’s frozen turbulence hypothesis to non-intrusive pipeline flow measurement using DAS technology. While previous studies have mainly focused on planar or open-channel turbulent flows, the examination of Taylor’s hypothesis in the context of fully developed turbulence in circular pipes offers novel insights with direct implications for practical engineering.

By analyzing the spatiotemporal evolution of turbulent kinetic energy, this study demonstrates that vortices within the pipe remain structurally coherent over time and are propagated at a speed closely related to the mean flow velocity. This supports the theoretical basis of Taylor’s hypothesis that turbulent structures can be “frozen” and advected along the streamwise direction under sufficient mean velocity. The resulting FK spectrum from the simulations further confirms the concentration of energy along a dominant diagonal line corresponding to the convection velocity, consistent with the prediction of the hypothesis.

The devised dispersion feature enhancement algorithm significantly improves the extraction of the main velocity-related energy lines in the FK spectrum. Based on normalized cross-correlation and a rotatable elliptical template, it enhances signal concentration and modulates the influence of spectral noise. These improvements lead to a more precise and consistent estimation of convection velocity, resulting in average velocity calculation errors below 3% in simulations and comparable accuracy in experiments.

The experimental system using DAS clearly demonstrates the feasibility of Taylor’s hypothesis in characterizing the average flow velocity in a circular pipe without requiring intrusive installations or external excitation sources. This method offers better stability and reliability, particularly in high-flow regions, compared to traditional flow-induced vibration (FIV) approaches. Unlike FIV, which relies on local statistics and is sensitive to boundary conditions, the proposed method fully exploits DAS’s distributed sensing capabilities.

However, some limitations exist. First, the current implementation is based on simulated turbulent pressure fluctuations, but DAS systems measure fiber axial strain—not pressure. A mapping between the two needs to be established through fluid–structure interaction modeling to bridge this gap and move the method closer to real-world deployment.

Second, the performance of the flow inversion process decreases under low-flow conditions due to environmental vibrations dominating the DAS signals. Future enhancements should consider machine learning-based signal interpretation models or spatiotemporal filtering techniques that improve signal-to-noise ratios and inversion robustness in low-fluid regimes.

These results and findings highlight the broader applicability of Taylor’s hypothesis to internal flow among cylindrical structures and pave the way for passive, real-time pipeline flow monitoring that does not rely on mechanical or active acoustic perturbations. Moving forward, the methodology can be extended in three potentially impactful directions:

Multiphase Flow Adaptability: While the current work focuses on single-phase flow, future studies should validate Taylor’s hypothesis in environments with gas–liquid or solid–liquid flow interactions. Turbulent structures in such flows may exhibit spatiotemporal instabilities, requiring adapted propagation models and signal compensation algorithms.

Geometric Applicability: Geometric factors such as pipe curvature, elliptical cross-sections, or flexible/partially supported structures may alter vortex dynamics and influence data interpretation. Changes in flow symmetry or secondary flow patterns necessitate DAS fiber layout optimization and model adaptation.

Deep Signal Enhancement: Deep learning models like spatiotemporal attention networks can be trained on DAS data to automatically extract multiscale vortex features and suppress noise under a variety of operational conditions. Transfer learning techniques may especially enhance low-flow measurement performance, where signal sensitivity is a known challenge.

This work provides both a methodological and theoretical foundation for the integration of DAS and vortex-based flow characterization. In summary, Taylor’s hypothesis shows significant potential in distributed non-intrusive flow measurement technology, especially when combined with advanced signal processing techniques. The proposed enhancement method lays the groundwork for further development in vacuum and aerospace applications, oil–gas transport pipelines, and intelligent flow monitoring systems.

## 6. Conclusions

This study confirms the applicability of Taylor’s frozen hypothesis in circular pipe turbulence through both LES and experimental validation using DAS technology. The proposed dispersion feature enhancement algorithm improves the detection accuracy of convection velocity by suppressing non-target directional interference. Results demonstrate that the derived average flow velocity has a relative error of less than 3% compared to the standard. This method shows advantages in high-flow conditions, outperforming traditional FIV-based approaches. These findings establish a new theoretical and practical framework for non-intrusive flow measurement using DAS and open new paths for real-time, spatially continuous flow monitoring in industrial applications.

## Figures and Tables

**Table 1:** Fluid domain structural dimensions and material property parameters.

Symbol	Meaning	Value	Unit
d	Fluid domain diameter	62	mm
l	Fluid domain length	0.2	m
$\rho_{f}$	Fluid density	997	kg/m^3^
ν	Kinematic viscosity	1.02 × 10^−6^	m^2^/s
Q	Quality flow rate	1.441~4.571	kg/s

**Table 2:** Relative deviation of convection velocity.

Flow Conditions (kg/s)	Average Flow Velocity/(m/s)	Convection Velocity/(m/s)	Relative Offset
1.441	0.4773	0.4701	−1.50%
2.517	0.8337	0.8164	−2.07%
3.007	0.9960	0.9951	−0.09%
3.544	1.1739	1.0490	−10.64%
4.058	1.3441	1.2113	−9.88%
4.571	1.5140	1.5606	3.08%

**Table 3:** Analysis of relative error in average speed calculation.

Average Flow Velocity/(m/s)	Convection Velocity/(m/s)	Calculate Average Speed/(m/s)	Relative Error
0.4773	0.4365	0.4821	1.01%
0.8337	0.7112	0.8501	1.97%
0.9960	0.7994	0.9683	2.78%
1.1739	0.9793	1.2093	3.00%
1.3441	1.0776	1.3409	0.24%
1.5140	1.1998	1.5046	0.62%

**Table 4:** Comparison and analysis of flow measurement and calculation results under different principles.

Standard Flow/(L/min)	Taylor’s Frozen		FIV Flow	
	Flow/(L/min)	Relative Error	Flow/(L/min)	Relative Error
86.46	103.43	19.63%	97.51	12.33%
151.02	134.61	10.86%	162.36	7.17%
180.42	168.57	6.57%	146.72	18.94%
212.64	223.11	4.92%	189.02	11.38%
243.48	254.91	4.70%	250.07	2.40%
274.26	263.64	3.87%	279.94	1.69%

## Data Availability

The theoretical simulation data and core algorithms (LES methodology, cross-correlation processing) are fully documented in the manuscript to ensure reproducibility. Due to laboratory policy and proprietary device limitations, raw experimental data (e.g., DAS spatiotemporal matrices, pressure signal time histories) cannot be hosted on public repositories. Researchers requiring specific datasets may request them from the corresponding author (Lei Liang, lianglei@whut.edu.cn) under confidentiality agreements.

## References

[1] Nour M., Hussain M.M. (2020). A Review of the Real-Time Monitoring of Fluid-Properties in Tubular Architectures for Industrial Applications. Sensors.

[2] Vadde A., Kadambi G.R., Channabasappa S. (2023). A review on non-invasive magnetic and electric field excited methods for flow characterisation of incompressible Newtonian low conductive liquids. J. Adv. Res. Fluid Mech. Therm. Sci..

[3] Evans R.P., Blotter J.D., Stephens A.G. (2004). Flow rate measurements using flow-induced pipe vibration. J. Fluids Eng..

[4] Biernacki P., Gmyrek S., Magiera W. (2023). Non-invasive Ultrasound Doppler Effect Based Method of Liquid Flow Velocity Estimation in Pipe. Arch. Acoust..

[5] Hu J., Wang Y. (2025). Wellbore single-phase fluid flow velocity calculation based on distributed acoustic sensing data. J. Geophys. Eng..

[6] Krivonogov A., Taranenko P.A., Khan A. (2024). Development of clump-on sonar flow meter using symmetry channel model. Int. J. Simul. Multidiscip. Des. Optim..

[7] Geng C., He G., Wang Y., Xu C., Lozano-Durán A., Wallace J.M. (2015). Taylor’s hypothesis in turbulent channel flow considered using a transport equation analysis. Phys. Fluids.

[8] Ng H.C.-H., Cregan H.L.F., Dodds J.M., Poole R.J., Dennis D.J.C. (2018). Partially filled pipes: Experiments in laminar and turbulent flow. J. Fluid Mech..

[9] Tardu S., Vezin P. (2005). On the Taylor hypothesis in forced unsteady wall flows. Exp. Fluids.

[10] Ambrogi F., Piomelli U., Rival D.E. (2023). Characterisation of unsteady separation in a turbulent boundary layer: Reynolds stresses and flow dynamics. J. Fluid Mech..

[11] Blyth T.S., Avramova M. (2017). Development and Implementation of CFD-Informed Models for the Advanced Subchannel Code CTF.

[12] Bayliss A., Gunzburger M., Turkel E. (1982). Boundary conditions for the numerical solution of elliptic equations in exterior regions. SIAM J. Appl. Math..

[13] Nieto J.J., Alvarez-Noriega N. (1996). Periodic boundary value problems for nonlinear first order ordinary differential equations. Acta Math. Hung..

[14] Rezaeiravesh S., Liefvendahl M. (2018). Effect of grid resolution on large eddy simulation of wall-bounded turbulence. Phys. Fluids.

[15] Cancilla N., Tamburini A., Tarantino A., Visconti S., Ciofalo M. (2022). Friction and Heat Transfer in Membrane Distillation Channels: An Experimental Study on Conventional and Novel Spacers. Membranes.

[16] Piomelli U., Liu C., Liu Z. (1997). Large-eddy simulations: Where we stand. Advances in DNS/LES.

[17] Eggels J.G.M., Unger F., Weiss M.H., Westerweel J., Adrian R.J., Friedrich R., Nieuwstadt F.T. (1994). Fully developed turbulent pipe flow: A comparison between direct numerical simulation and experiment. J. Fluid Mech..

[18] Verma M.K., Kumar A. (2014). Sweeping effect and Taylor’s hypothesis via correlation function. arXiv.

[19] Huang Y., Biferale L., Calzavarini E., Sun C., Toschi F. (2013). Lagrangian single-particle turbulent statistics through the Hilbert-Huang transform. Phys. Rev. E—Stat. Nonlinear Soft Matter Phys..

